# Antimicrobial Resistance Incidence and Risk Factors among *Helicobacter pylori*–Infected Persons, United States

**DOI:** 10.3201/eid1006.030744

**Published:** 2004-06

**Authors:** William M. Duck, Jeremy Sobel, Janet M. Pruckler, Qunsheng Song, David Swerdlow, Cindy Friedman, Alana Sulka, Balasubra Swaminathan, Tom Taylor, Mike Hoekstra, Patricia Griffin, Duane Smoot, Rick Peek, David C. Metz, Peter B. Bloom, Steven Goldschmid, Julie Parsonnet, George Triadafilopoulos, Guillermo I. Perez-Perez, Nimish Vakil, Peter Ernst, Steve Czinn, Donald Dunne, Ben D. Gold

**Affiliations:** * Centers for Disease Control and Prevention, Atlanta, Georgia, USA;; † Howard University Medical Center, Washington, DC, USA;; ‡ Vanderbilt University Medical Center, Nashville, Tennessee, USA;; § University of Pennsylvania Medical Center, Philadelphia, Pennsylvania, USA;; ¶ Atlanta Veterans Affairs Medical Center, Atlanta, Georgia, USA;; # Stanford University Medical Center, Palo Alto, California, USA;; ** New York University School of Medicine, New York, New York, USA;; †† Sinai Samaritan Medical Center, Milwaukee, Wisconsin, USA;; ‡‡ University of Virginia, Charlottesville, Virginia, USA;; §§ University Hospitals of Cleveland, Cleveland, Ohio, USA;; ¶¶ Indiana University Medical Center, Indianapolis, Indiana, USA

**Keywords:** *Helicobacter pylori*, antimicrobial resistance, risk factors

## Abstract

*Helicobacter pylori* is the primary cause of peptic ulcer disease and an etiologic agent in the development of gastric cancer. *H. pylori* infection is curable with regimens of multiple antimicrobial agents, and antimicrobial resistance is a leading cause of treatment failure. The *Helicobacter pylori* Antimicrobial Resistance Monitoring Program (HARP) is a prospective, multicenter U.S. network that tracks national incidence rates of *H. pylori* antimicrobial resistance. Of 347 clinical *H. pylori* isolates collected from December 1998 through 2002, 101 (29.1%) were resistant to one antimicrobial agent, and 17 (5%) were resistant to two or more antimicrobial agents. Eighty-seven (25.1%) isolates were resistant to metronidazole, 45 (12.9%) to clarithromycin, and 3 (0.9%) to amoxicillin. On multivariate analysis, black race was the only significant risk factor (p < 0.01, hazard ratio 2.04) for infection with a resistant *H. pylori* strain. Formulating pretreatment screening strategies or providing alternative therapeutic regimens for high-risk populations may be important for future clinical practice.

The prevalence of *Helicobacter pylori* infection worldwide is approximately 50% (*1*), as high as 80%–90% in developing countries, and ≈35%–40% in the United States (*1*). Approximately 20% of persons infected with *H. pylori* develop related gastroduodenal disorders during their lifetime (*1*). *H. pylori* is an etiologic agent of peptic ulcer disease, primary gastritis, gastric mucosa-associated lymphoid-tissue lymphoma, and gastric adenocarcinoma (*2*). The annual incidence of *H. pylori* infection is ≈4%–15% in developing countries, compared with approximately 0.5% in industrialized countries (*3*). Documented risk factors include low socioeconomic status, overcrowding, poor sanitation or hygiene, and living in a developing country (*2*).

Eradication therapy of symptomatic *H. pylori* infection substantially reduces the recurrence of associated gastroduodenal diseases. Therapy entails complicated regimens of several antimicrobial agents for at least 2 weeks. In general, triple therapy regimens usually entail two of the following antimicrobial agents: metronidazole, amoxicillin, tetracycline, or clarithromycin in combination with a proton pump inhibitor or bismuth (*4*). The most common causes of treatment failure are patient noncompliance and antimicrobial resistance of the infecting *H. pylori* strain (*5*). Quadruple regimens are used as a salvage therapy when triple therapy regimens have failed (*4*). Pretreatment resistance of *H. pylori* has been reported to compromise the efficacy of treatment. For example, therapy regimens containing metronidazole and clarithromycin fail in as many as 38% and 55% of cases, respectively, when used to treat infection with an organism resistant to one of these antimicrobial agents (*6*). In addition, published data are lacking that describe the effect conferred by polymicrobial resistance on eradication success.

The *Helicobacter pylori* Antimicrobial Resistance Monitoring Project (HARP) is a prospective, longitudinal network monitoring ongoing national and regional trends of antimicrobial resistance in *H. pylori* isolates in the United States. The network is positioned to document emerging resistance and to assist physicians in formulating therapy recommendations. We present the results of the antimicrobial resistance monitoring and risk factor analysis from data collected prospectively from 1998 through 2002.

## Materials and Methods

During the course of the study, HARP consisted of 11 hospital study sites across the United States. The first five patients who sought treatment each month for esophago-gastro duodenal endoscopy (EGD) as part of treatment for *H. pylori* infection, confirmed by gastric biopsy, were enrolled at each site. When fewer than five eligible study participants were seen at a study center, those eligible were enrolled. Written, signed consent was obtained from all participants or their guardians. Culture of biopsied material for *H. pylori* was performed at the study site or at the Atlanta Veterans Affairs Hospital. If primary isolation was performed at the HARP study site, isolates were placed in trypticase soy broth (TSB) supplemented with glycerol and stored at –70°C. Isolates were sent to the Centers for Disease Control and Prevention for antimicrobial resistance testing. Samples forwarded to the Atlanta Veterans Affairs Hospital for isolation were packed in 6 pounds of dry ice and maintained at –70°C to –80°C until cultured (up to 1 month). Each specimen was accompanied by a standard case report form containing 145 variables of demographic, clinical, and epidemiologic information.

### Laboratory Procedures

Isolates were cultured on heart infusion agar (HIA) with 5% rabbit blood at 37°C in a microaerobic atmosphere (10% CO_2_, 85% N_2_, 5% O_2_) for a minimum of 72 hours or until appearance of *H. pylori* colonies. Gastric biopsy specimens were homogenized by using a micropestle in a microcentrifuge tube, and the homogenates were added to HIA with 5% rabbit’s blood and Skirrow media and incubated at 37°C microaerobically for up to 2 weeks. Presumptive *H. pylori* colonies were subcultured for further testing. Isolates were confirmed as *H. pylori* if they demonstrated typical morphologic features by dark-field microscopy and if urease, oxidase, and catalase activities were detected. Also, a polymerase chain reaction amplification assay that used primers targeted at the *ure*A gene provided confirmation (*7*). Antimicrobial susceptibility testing was performed by the agar dilution method in accordance with National Committee for Clinical Laboratory Standards (NCCLS) protocols, except that defibrinated rabbit blood was used instead of aged sheep blood (*8*). For antimicrobial agents without an NCCLS-recommended breakpoint, breakpoints were selected after a review of the literature.

### Data Collection and Analysis

Case report forms and laboratory data were entered into a MS Access 2000 database and analyzed by using SAS, version 8.2 (SAS Institute, Cary, NC). We defined a resistant case to be an HARP isolate that demonstrated any detectable resistance to metronidazole, clarithromycin, tetracycline, or amoxicillin. HARP study sites were assigned to the following four regions: Northeast (Washington, DC; New York, NY; and Philadelphia, PA), South (Atlanta, GA; Nashville, TN; and Galveston, TX), Midwest (Detroit, MI; Cleveland, OH; Indianapolis, IN; and Milwaukee, WI), and West (Palo Alto, CA). Differences between patients infected with resistant and susceptible strains of *H. pylori* were assessed by using the Fisher exact test, Mantel-Haenszel chi-square, or Student *t* test, as appropriate. A one-way analysis of variance (ANOVA) was used to determine antimicrobial resistance rate trends for clarithromycin and metronidazole. When the difference in the number of study participants that harbored a resistant *H. pylori* infection and those who did not for a given risk factor was <2, we chose to exclude the risk factor from further analysis because of the possibility of arriving at a spurious association caused by small cell-size variations. This method enhanced the statistical reliability of all possible exposure and resistance associations considered for multivariate modeling. Risk factors that met these criteria were subjected to a chi-square score model selection procedure to obtain the most significant and stable multivariate model. Multivariate analyses were performed by using logistic regression to assess the independent association of identified risk factors with any detectable antimicrobial resistance using two different models.

The first logistic regression model assessed significant univariate risk factors for antimicrobial resistance. A second logistic regression model was created that we conditioned on geographic location of HARP site, previous antimicrobial treatment for *H. pylori* infection, and antacid use to resolve colinearity issues with race.

## Results

Of 317 enrolled HARP study participants for whom complete demographic information was available, 205 (65%) were male, 116 (37%) were white, 172 (54%) were black, 14 (4%) were Asian, 3 (1%) were Native American, and 12 (4%) were of other ethnic backgrounds. The median age was 57 years (range 3–94 years). Among males, 97 (31%) were white, 90 (28%) were black, and 9 (3%) were Asian, 1 (0.3%) was Native American, and 8 (3%) were of other ethnic backgrounds. Among females, 19 (6%) were white, 82 (26%) were black, 5 (2%) were Asian, 2 (0.6%) were Native American, and 4 (1%) were of other ethnic backgrounds. The most common endoscopic diagnoses for conditions of HARP participants were gastric erosions, gastritis, duodenal and gastric ulcers, and esophagitis (Table 1). *H. pylori* resistance was not statistically associated with endoscopic findings.

**Table 1:** Endoscopy findings on patients enrolled in the *Helicobacter pylori* Antimicrobial Resistance Monitoring Project, 1998–2002^a^

Endoscopic diagnosis	No. of patients (%)
Stomach erosion	88 (27.0)
Gastritis	36 (11.0)
Duodenal ulcers	26 (8.0)
Esophagitis	26 (8.0)
Gastric ulcer	26 (8.0)
Duodenal erosion	24 (7.0)
Barrett esophagus	9 (3.0)
Nodularity	8 (2.0)
Stomach tumor	4 (1.0)
Bleeding^b^	1 (0.3)
Other diagnosis	27 (8.0)

^a^N = 331; *H. pylori* resistance is not associated with disease phenotype. ^b^Cause not specified.

Among 347 *H. pylori* isolates submitted to HARP from 1998 to 2002, 118 (34%) were resistant to >1 antimicrobial agent, 101 (29%) *H. pylori* isolates were resistant to one agent only, and 17 (5%) *H. pylori* isolates were resistant to more than one antimicrobial agent. Three isolates were resistant to amoxicillin (1%), 45 were resistant to clarithromycin (13%), 87 were resistant to metronidazole (25%), and no isolate was found to be resistant to tetracycline. Multiple-agent resistance was observed for clarithromycin and amoxicillin (1 isolate, 0.3%) and clarithromycin and metronidazole (16 isolates, 5%). A test of trend showed no significant trend for resistance to metronidazole, but a significant trend (R^2^ = 0.76) was noted for a decline in resistance to clarithromycin during the study period (Figure).

**Figure:**
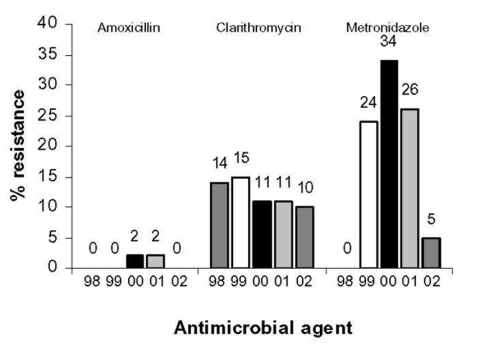
Resistance of *Helicobacter pylori* isolates submitted to the *Helicobacter pylori* Antimicrobial Resistance Monitoring Project, 1998–2002 (N = 347). 1998, n = 7 isolates; 1999, n = 137 isolates; 2000, n = 117 isolates; 2001, n = 47 isolates; 2002, n = 39 isolates. Resistance to tetracycline was found in 0% of isolates during each year of the monitoring project.

Most isolates were submitted by centers in the Northeast region, followed by the South, Midwest, and the West. Submitted *H. pylori* isolates from the Northeast region had the highest frequency of single- and dual-agent resistance (Table 2). The Midwest region submitted *H. pylori* isolates with the second-highest single agent resistance rate, while the southern region had the second-highest dual agent resistance rate. The highest proportion of *H. pylori* isolates resistant to clarithromycin, metronidazole, and amoxicillin was in the Northeast (Table 2).

**Table 2:** Resistance of HARP isolates by region^a^

Region	No. isolates	Amoxicillin No. (%)	Tetracycline No. (%)	Clarithromycin No. (%)	Metronidazole No. (%)	Resistant to 1 agent No. (%)	Resistant to >1 agent No. (%)	Resistant to >1 agent No. (%)
Northeast	156	2 (1)	0	23 (15)	47 (30)	54 (35)	9 (6)	63 (40)
South	92	1 (1)	0	13 (14)	13 (14)	19 (21)	4 (4)	23 (25)
West	24	0	0	1 (4)	4 (17)	3 (13)	1 (4)	4 (17)
Midwest	75	0	0	8 (11)	23 (31)	25 (33)	3 (4)	28 (37)
Total	347	3	0	45	87	101	17	118

^a^N = 347; HARP, *Helicobacter pylori* Antimicrobial Resistance Monitoring Project.

The four sites reporting the highest proportion of isolates resistant to one agent were Indianapolis, Indiana; Galveston, Texas; Philadelphia, Pennsylvania; and Washington, DC (Table 3). Dual agent resistance was most prevalent in Detroit, Michigan. Indianapolis had the highest overall resistance: 50% of all isolates submitted were resistant to at least one agent. The Atlanta site reported the highest proportion of clarithromycin-resistant isolates (Table 3). The highest proportion of metronidazole resistance was reported from the Indianapolis site. Isolates resistant to amoxicillin were found in Nashville, Tennessee, Philadelphia, and Washington, DC.

**Table 3:** Resistance of HARP *H. pylori* isolates by site^a^

Location	No. isolates	Amoxicillin No. (%)	Tetracycline No. (%)	Clarithromycin No. (%)	Metronidazole No. (%)	Resistant to 1 agent No. (%)	Resistant to >1 agent No. (%)	Resistant to >1 agent No. (%)
Atlanta, GA	37	0	0	7 (19)	5 (14)	4 (11)	4 (11)	8 (22)
Nashville, TN	45	1 (2)	0	5 (11)	5 (11)	11 (24)	0	11 (24)
Galveston, TX	10	0	0	1 (10)	3 (30)	4 (40)	0	4 (40)
Philadelphia, PA	41	1 (2)	0	6 (15)	15 (37)	16 (39)	3 (7)	19 (46)
Washington, DC	93	1 (1)	0	15 (16)	31 (33)	35 (38)	6 (7)	41 (44)
Indianapolis, IN	36	0	0	3 (8)	15 (42)	18 (50)	0	18 (50)
Detroit, MI	18	0	0	3 (17)	4 (22)	3 (17)	2 (11)	5 (28)
Cleveland, OH	6	0	0	0	1 (17)	1 (17)	0	1 (17)
Milwaukee, WI	15	0	0	2 (13)	3 (20)	3 (20)	1 (7)	4 (27)
Stanford, CA	24	0	0	1 (4)	4 (17)	3 (13)	1 (4)	4 (17)
New York, NY	22	0	0	2 (9)	1 (5)	3 (14)	0	3 (14)

^a^N = 347; HARP, *Helicobacter pylori* Antimicrobial Resistance Monitoring Project.

Demographic, clinical, and epidemiologic data were analyzed for association with infection by a resistant *H. pylori* strain. Results of univariate analysis of risk factors for antimicrobial resistance are summarized in Table 4. On univariate analysis, significant risk factors for *H. pylori* resistance included past treatment of *H. pylori* infection with clarithromycin, treatment of past *H. pylori* infection for a second time in the past 5 years, treatment of past *H. pylori* infection with clarithromycin more than once, and treatment of past *H. pylori* infection with a proton pump inhibitor more than once. No protective factors for resistant *H. pylori* resistance approached significance.

**Table 4:** Univariate analysis of risk factors for resistance to >1 antimicrobial agent among *Helicobacter pylori* isolates submitted to HARP, 1998–2002^a^

Risk factor	Proportion of patients harboring resistant *H. pylori* isolate		
	Exposed, no. (%)	Unexposed, no. (%)	Odds ratio (95% CI)
Zantac use 12 mo before EGD	25 (48)	89 (32)	2.0 (1.1–3.6)^b^
Tums use 12 mo before EGD	19 (51)	95 (32)	2.2 (1.1–4.4)^b^
Tums use 30 d before EGD	12 (55)	102 (33)	2.4 (>1.0–5.8)^b^
Mylanta use 12 mo before EGD	4 (15)	110 (36)	0.3 (0.1–1.0)
Took antibiotic 12 mo before EGD	29 (43)	68 (34)	1.5 (0.9–2.6)
Past treatment of *H. pylori* infection with:			
PPI	5 (71)	109 (34)	4.9 (1.0–26)
Clarithromycin	5 (83)	109 (33)	9.9 (1.1–86)
Treatment for *H. pylori* a second time in the past 5 years	11 (65)	107 (32)	3.8 (1.4–11)
Treatment of *H. pylori* infection more than once with			
PPI	8 (80)	106 (33)	8.1 (1.7–39)
Clarithromycin	8 (80)	106 (33)	8.1 (1.7–39)
Age >57 years	50 (31)	68 (37)	0.8 (0.5–1.2)
Race/ethnicity			
Black	67 (39)	51 (29)	1.6 (1.0–2.4)
Hispanic	2 (11)	116 (35)	0.2 (0.1–1.0)
Male sex	65 (31)	47 (42)	0.6 (0.4–1.0)
HARP site			
New York, NY	3 (14)	115 (35)	0.3 (0.1–1.0)
Palo Alto, CA	4 (17)	114 (35)	0.4 (0.1–1.1)
Atlanta, GA	8 (22)	110 (36)	0.5 (0.2–1.1)
Nashville, TN	11 (24)	107 (35)	0.6 (0.3–1.2)

^a^N = 347; HARP, *Helicobacter pylori* Antimicrobial Resistance Monitoring Project; EGD, esophagogastro-duodenal endoscopy; PPI, proton pump inhibitor; CI, confidence interval. ^b^These risk factors were not considered for multivariate analysis because of small cell-size differences between subjects with a resistant *H. pylori* infection and those without.

On multivariate analysis, the most significant risk factor for *H. pylori* resistance was previous treatment of *H. pylori* infection within the past 5 years (Table 5). Protective factors for *H. pylori* infection were treatment with an antacid (Mylanta) and submission of specimens from New York, Palo Alto, California, Atlanta, or Nashville. However, a high degree of colinearity was found between geographic location and race in this model. For example, 84% of Washington, DC, participants were black, compared to 20% of participants from Nashville. Accordingly, a second multivariate model was constructed in which geographic location was controlled by conditioning. In this model, black race was the only risk factor significantly associated with infection with a resistant *H. pylori* strain (Table 6).

**Table 5:** Phase I multivariate analysis of risk factors for resistance to >1 antimicrobial agent among *H. pylori* isolates submitted to HARP, 1998–2002^a^

Exposure	Odds ratio (95% CI)
Patient treated another time for infection	6.0 (2.0–20)
Took Mylanta in last 12 mo	0.3 (0.1–0.8)
HARP site	
New York, NY	0.2 (0.1–0.7)
Palo Alto, CA	0.2 (0.1–0.7)
Atlanta, GA	0.2 (0.1–0.6)
Nashville, TN	0.4 (0.2–0.9)

^a^N = 347; HARP, *Helicobacter pylori* Antimicrobial Resistance Monitoring Project; CI, confidence interval.

**Table 6:** Phase II stratified multivariate analysis of risk factors for resistance to >1 antimicrobial agent among *H. pylori* isolates submitted to HARP, 1998–2002^a^

Risk factor	Hazard ratio (95% CI)
Black race	2.1 (1.1–3.8)
Age >57 y	0.6 (0.3–1.1)
Took antibiotics 12 mo before esphagogastro- duodenal endoscopy	1.9 (0.9–3.7)

^a^N = 235; HARP, *Helicobacter pylori* Antimicrobial Resistance Project; CI, confidence interval.

## Discussion

The emergence of antimicrobial resistance in *H. pylori* represents a serious public health challenge because of the prevalence of infection and incidence of severe sequelae. A 12-fold increase in resistance to antimicrobial agents such as clarithromycin has been documented in cases in which antimicrobial therapy regimens do not eliminate *H. pylori* (*9*). Once resistance to clarithromycin appears, cross-resistance to all other macrolides also occurs, which eliminates the potential for their use in salvage therapies. In the past 5 years, amoxicillin and tetracycline resistance has also been described (*6**,**10*).

*H. pylori* resistance is amply documented in a variety of study populations. However, most published studies have been based on cross-sectional designs, not prospective population-based surveillance of resistance. A meta-analysis of 20 nationwide clinical trials estimated *H. pylori* resistance to clarithromycin, metronidazole, and amoxicillin to be 10.1%, 36.9%, and 1.4%, respectively (*6*). These findings were also reflected in smaller single-site studies (*11*). Resistance rates may be higher in pediatric populations; for example, a study in Michigan demonstrated that clarithromycin resistance in pediatric *H. pylori* strains was 2.5 times higher than that reported in adults in the same region and elsewhere in the United States (*12*).

Single-site and multicenter surveillance network studies in Britain, Croatia, the Czech Republic, Turkey, Greece, Poland, Russia, Slovenia, Turkey, Estonia, Italy, Bulgaria, Germany, and Belgium have observed primary metronidazole and clarithromycin resistance with rates as high as 40.3% and 10.6%, respectively (*13**–**17*). A large prospective surveillance project in northeastern Germany showed primary metronidazole and clarithromycin resistance rates of 26.2% and 2.2%, respectively, in 1,644 clinical *H. pylori* isolates collected from 1995 to 2000 (*17*). High resistance rates to metronidazole and clarithromycin have been reported in Portugal, Brazil, Hong Kong, Saudia Arabia, and Lebanon (*18**–**22*). Overall, *H. pylori* resistance to metronidazole among single and multicenter surveillance networks is greater than all other resistant antimicrobial agents combined.

HARP is the only multicenter network providing ongoing prospective antimicrobial resistance and associated risk factor data for *H. pylori* in North America. The first 4 years of data show that resistance to antimicrobial drugs commonly used to treat *H. pylori* infections is widespread, though rates varied from year to year; this finding was true for both clarithromycin and metronidazole. We speculate that the precipitous decrease in metronidazole resistance in 2002 could be attributed to the reduction in isolates received by HARP, which limited the power and representiveness of our sample.

We performed a two-phased multivariate analysis on risk factors significantly associated with resistance on univariate analysis. In the first-phase, multivariate model, antimicrobial resistance was significantly associated with previous antimicrobial treatment for *H. pylori*, and antacid use was protective. However, in this model, geographic location was highly collinear with race, which necessitated the second-phase multivariate model, in which the contribution of race was assessed by conditioning on geographic location, previous antimicrobial treatment for *H. pylori* infection, and antacid use. This model clearly demonstrated an association between black race and infection with an antimicrobial-resistant *H. pylori*. This association may indicate persistent transmission of resistant strains or in vivo induction of resistance. The association between *H. pylori* infection and blacks in the United States has been amply documented (*2**,**3*). However, the association between incidence of *H. pylori* resistance and black race is new. The biologically plausible association between previous antimicrobial use and resistance approached significance in the second-phase model; however, many study participants could not recall whether they were previously treated. This model does not rule out an independent association between geographic location and resistance. Enrollment of additional participants in HARP may sufficiently enhance the statistical power of the study to show such an association, while enrollment in sites serving a more racially diverse population may help clarify the contributions of geographic location as a risk factor for resistant infection.

This study has several limitations. While drawn from various regions in the continental United States, the 11 HARP sites represent a convenience sample of academic medical centers that is not truly representative of the U.S. population. Some participating sites enrolled more patients per month than others, so that cumulative data are biased towards individual study sites. Most of the participating hospital sites serve adult populations, and thus the results largely reflect resistance incidence towards adult patients. Patients undergoing endoscopy for *H. pylori*-related illness constitute a minority of infected persons, whose clinical condition and *H. pylori*-resistance status may not be representative of the total population with underlying *H. pylori* infection. Although this trend has not been documented, many public health analysts suspect that the proportion of *H. pylori* infections diagnosed and treated in academic medical centers is decreasing compared with those in community practices. We suspect that this trend may have accounted for the reduced number of isolates submitted to HARP over time. Although these limitations primarily affected our sample size and how the sample represented the population, they did not invalidate the epidemiologic significance of the results generated from this study.

Given the limited population-based surveillance data on *H. pylori* resistance, HARP offers the best available data on *H. pylori* resistance in the United States. The accuracy and usefulness of this prospective, multicenter network can be enhanced in a number of ways. Increasing the number of HARP sites will improve geographic and demographic representation. Given the fact that only 12 study participants <12 years of age were included, enrollment of pediatric-care sites will improve the age representation of the HARP cohort. This feature is particularly important considering the increasing number of reports that describe higher resistance rates in pediatric strains compared to those isolated from adults, particularly resistance to macrolides (*12**,**23*). Enrolling study participants in community practices will provide data on resistance rates in *H. pylori* isolates from infected persons not treated at academic medical centers, a population in which resistance rates in *H. pylori* are unknown and may differ significantly from the current HARP cohort. Future HARP findings may serve as the basis for specific therapeutic recommendations and pretreatment antimicrobial susceptibility testing in high-risk populations.

In summary, we have shown that antimicrobial resistance in clinical *H. pylori* isolates is extensive, that it varies from year to year, and that resistant isolates are more common among blacks. Ongoing, prospective surveillance of *H. pylori* resistance is essential to assure [or ensure?] that appropriate data are available to guide the choice of therapy, particularly in high-risk populations.
